# Metabolic dysregulation in obese women and the carcinogenesis of gynecological tumors: A review

**DOI:** 10.17305/bb.2024.10508

**Published:** 2024-08-01

**Authors:** Dragana Tomić Naglić, Aljoša Mandić, Andrijana Milankov, Slađana Pejaković, Stefan Janičić, Nikolina Vuković, Ivana Bajkin, Tijana Ičin, Mia Manojlović, Edita Stokić

**Affiliations:** 1 University of Novi Sad, Faculty of Medicine in Novi Sad, Novi Sad, Serbia; 2 Diabetes and Metabolic Disorders, Clinic for Endocrinology, Clinical Center of Vojvodina, Novi Sad, Serbia; 3 Institute of Oncology of Vojvodina, Sremska Kamenica, Serbia

**Keywords:** Obesity, cancer, gynecological malignancies, metabolic disorders.

## Abstract

Obesity is a significant health issue associated with increased cancer risks, including gynecological malignancies. The worldwide rise in obesity rates is significantly impacting both cancer development and treatment outcomes. Adipose tissue plays a crucial role in metabolism, secreting various substances that can influence cancer formation. In obese individuals, dysfunctional adipose tissue can contribute to cancer development through inflammation, insulin resistance, hormonal changes, and abnormal cholesterol metabolism. Studies have shown a strong correlation between obesity and gynecological cancers, particularly endometrial and breast cancers. Obesity not only increases the risk of developing these cancers but is also associated with poorer outcomes. Additionally, obesity affects the perioperative management of gynecological cancers, requiring specialized care due to increased complications and resistance to therapy. Treatment strategies for managing metabolic dysregulation in patients with gynecological cancers include weight management, statin therapy, and insulin-sensitizing medications. Emerging studies suggest that interventions like intermittent fasting and caloric restriction may enhance the effectiveness of cancer treatments. Furthermore, targeting cholesterol metabolism, such as with statins or proprotein convertase subtilisin/kexin type 9 (PCSK9) inhibitors, shows potential in cancer therapy. In conclusion, addressing metabolic issues, particularly obesity, is crucial in preventing and treating gynecological malignancies. Personalized approaches focusing on weight management and metabolic reprogramming may improve outcomes in these patients.

## Introduction

According to the definition of the World Health Organization, obesity is an excessive presence of fat mass in the body composition, which leads to impairment of health and the development of complications. At the global level, it was estimated that in 2022, about 2.5 billion adults were overweight or obese. Also, it is known that obesity most often causes mass noncontagious diseases, such as cardiovascular diseases, dyslipidemia, and diabetes, but also some malignancies, of which endometrial cancer, breast cancer, ovarian, kidney, and colon cancers are particularly prominent [1, 2].

It is well known that adipose tissue is an energy depot and a metabolically active tissue. It secretes numerous adipokines and cytokines that influence metabolic regulation. The metabolic activity of adipose tissue largely depends on its localization. Adipose tissue around visceral organs leads to metabolic disorders, while subcutaneous adipose tissue and adipose tissue distributed in the lower extremities are more responsible for musculoskeletal disorders and venous stasis [3]. In addition, adipose tissue also leads to the activation of cancer signaling pathways (phosphoinositide 3-kinases [PI3K], mitogen-activated protein kinase [MAPK], IKK, signal transducer and activator of transcription 3 [STAT3]). Through the phenomenon of adipocyte-cancer cell crosstalk, in obese individuals with malignancies, morphological and functional changes occur in already dysfunctional adipose tissue [4].

Visceral obesity is frequently followed by metabolic syndrome, although it is not a mandatory diagnostic criteria for this cluster of disorders. Individual components of metabolic syndrome (increased waist circumference [WC], hypertriglyceridemia, fasting hyperglycemia, hypertension, and low levels of HDL cholesterol) represent independent risk factors for the development of carcinoma. For these reasons, modern obesitology and oncology require prevention, diagnosis, and treatment of each individual mentioned factor, independent of the presence of obesity and metabolic syndrome [5, 6].

Apart from the fact that dysfunctional adipose tissue participates in carcinogenesis, obesity also significantly affects the perioperative treatment of gynecological malignancies. Caring for these patients requires expensive instruments and preparation for surgery by a multidisciplinary team due to the presence of numerous comorbidities and more frequent postoperative complications in the form of thromboembolism or more frequent wound infections. Furthermore, obese patients are often resistant to neoadjuvant therapy, which reduces overall survival [7]. In addition to smoking, obesity is considered one of the most critical risk factors for the development of cancer, which can be modified [8].

An extensive prospective analysis of 245,009 female patients pointed out that traditional nutritional indicators, body mass index (BMI), WC, and hip-to-waist ratio (WHR) may be associated with an increased risk of developing endometrial cancer [9].

Among gynecological cancers, breast cancer and endometrial cancer are most commonly associated with obesity. Recent studies have suggested a positive correlation between BMI and disease recurrence and overall survival [10].

A strong correlation between BMI and the incidence of endometrial cancer has been demonstrated. It is believed that more than 50% of patients with newly diagnosed endometrial cancer are obese. It was observed that mortality in these patients is twice higher for those with a BMI of 30–34.9 kg/m^2^, while for extremely obese patients (BMI over 40 kg/m^2^), the mortality is six times higher compared to people with normal body weight [10]. It was also observed that the existence of metabolic syndrome (without the mandatory presence of obesity) doubles the risk for the development of endometrial cancer in both premenopausal and postmenopausal women [7]. Furthermore, a recent study emphasized the importance of evaluating comorbidities in patients with endometrial cancer, as they are highly prevalent and influence survival outcomes. Thus, scoring systems have been developed to effectively summarize the impact of comorbidities and age on oncologic outcomes. One such system is the Age-adjusted Charlson Comorbidity Index, where a score of 3 or higher indicates a significant risk for mortality, a high risk of disease recurrence, and aggressive tumor characteristics. The same study highlighted the significance of diabetes, which emerged as the sole independent predictor of cause-specific survival [11].

Obese individuals with endometrial carcinoma also have poorer disease outcomes. Patients with a BMI of 30–34.9 kg/m^2^, compared to those who are normally nourished, have a 2.53 times higher risk of mortality, and for extremely obese patients, this risk increases to 6.25 times [12].

As there is a global obesity pandemic, with a continuous increase in the incidence of obesity among women, it is predicted that the incidence of endometrial carcinoma will also rise. By the year 2030, it is expected to be a 55% increase compared to 2010 (42.13/100,000 women) [13].

Meta-analyses have shown that BMI is an independent and positive indicator of breast cancer risk in postmenopausal age for obese women, the risk is higher by 82%. [14]. In postmenopausal women, a strong correlation has been observed between the increase in BMI and the incidence of developing breast cancer with positive expression of estrogen receptor alpha (ERα) and progesterone receptor (PR). The clearest association between obesity and breast cancer has been described in the luminal B molecular subtype, which is characterized by an aggressive course of the disease, frequent remissions, and a high mortality rate. For other subtypes of breast cancer in obese women, the literature is currently controversial [15].

The association of obesity with only some histological forms of ovarian cancer has been proven: (A) There is no connection between high-grade serous and high-grade endometrioid adenocarcinomas, (B) A weak association was verified with mucinous (17%), clear-cell tumors (19%), low-grade serous (13%), and low-grade endometrial cancers (20%), (C) The strongest association was detected with serous borderline tumors (20%–25%, with an increased risk of 9%–11% for every 5 kg change in body weight) [10].

Collectively assessed based on current studies, with every increase in BMI by one unit, the risk of developing ovarian carcinoma increases by 6% [12].

Regarding metabolic disorders in ovarian carcinomas, hyperglycemia and aerobic glucose metabolism are particularly notable, leading to the activation of the FBN1/VEGFR2/STAT2 pathway, consequently resulting in resistance to chemotherapy and stimulation of angiogenesis in tumor cells [16]. For this particular reason, there is a need, during the treatment of ovarian carcinomas, to concurrently undertake metabolic reprogramming, namely, the enhancement of anaerobic glycolysis, which would increase the sensitivity of the carcinoma to neoadjuvant therapy. The increased sensitivity of cancer cells during anaerobic glycolysis is explained by the tumor’s inadaptability to this type of metabolism.

Cervical cancer is more common in the form of adenocarcinoma in obese patients. It is believed that more frequent HPV infections and vaginal dysbiosis contribute to the carcinogenesis of this disease in obese patients [7]. On the other hand, HPV infection activates a series of aerobic glycolysis pathways, which are characteristic for the metabolism of cancer cells (elevated synthesis of lactate dehydrogenase A) [17].

Overall, each increase in body weight by 5 kg increases the relative risk of developing cancer in any location by 1,11. Maintaining a stable body weight is crucial, especially in postmenopausal women. The relative risk at that age for every 5 kg gain in body weight increases by 1,39 for endometrial cancer in women who have not used hormone replacement therapy (HRT). In contrast, in women with previous use of HRT, the risk of developing endometrial cancer is significantly lower 1,09. At the same time, 1,13 is the risk for women who are not obese and have not used HRT to develop ovarian cancer [7]. It is certainly apostrophized that obesity is an important risk factor for the development of gynecological malignancies, much more significant than the use of HRT.

## Metabolic dysregulation and carcinogenesis

Comprehensive studies in oncology and obesitology, published in recent years, have helped us understand, at the molecular level, carcinogenesis in obese patients [18].

The mechanism by which obesity is associated with carcinogenesis is multidimensional. Obesity is considered a disease with a low degree of inflammation. Due to the lipotoxicity in adipose tissue, there is damage to the cellular membrane, consequently leading to cellular injury, and exacerbating the inflammatory process. As a response, adipose tissue infiltration occurs primarily by the anti-inflammatory M2 subtype of macrophages, but there is also infiltration of proinflammatory macrophages of the M1 subpopulation. The M1 subfraction of macrophages secretes the inflammatory cytokines interleukin-6 (IL-6), tumor necrosis factor α (TNF-α), and interleukin 1β (IL-1β), activating the nuclear factor-κB (NFκB) and c-Jun N-terminal kinase (JNK) signaling pathway and leading to oxidative DNA damage and, thus, carcinogenesis [19].

Signaling pathways of inflammatory cytokines are listed in Table 1. Although it is noted that IL-6, through the JAK-STAT family signaling pathway, is supposed to have a protective effect by inhibiting proliferation, its synergistic action with EGFR demonstrates carcinogenic potential through the specific JAK2-STAT3 pathway along with other mentioned signaling cascades [20].

**Table 1:** Signal pathways of inflammatory cytokines and their role in cancerogenesis

**Cytokine**	**The pathway**	**Effect**
IL-6	*↑* JAK-STAT	Proliferation inhibition
IL-6+EGFR	*↑* JAK2-STAT3 *↑*PI3K/AKT *↑*ERK	Angiogenesis stimulation Apoptosis inhibition
TNF-α	Modulation of NF-κB, ERK1/2	Angiogenesis stimulation

IL-6: Interleukin 6; EGFR: Epidermal growth factor receptor; JAK-STAT: Janus kinase/signal transducer and activator of transcription; PI3K/AKT: Phosphatidylinositol 3-kinase/protein kinase B; ERK: Extracellular signal-regulated kinase; TNF-α: Tumor necrosis factor alpha; NF-κB: Nuclear factor kappa-light-chain-enhancer of activated B cells.

The previously mentioned lipotoxicity interferes with insulin signaling pathways, leading to insulin resistance and elevated insulin levels in the circulation. Hyperinsulinemia stimulates the growth of various organs and tissues, most likely through overexpression of the receptor for insulin-like growth factor-1 (IGF-1). This sensitizes cells to the proliferative effect of insulin, contributing primarily to endometrial hyperplasia. In addition, hyperinsulinemia leads to increased production of IGF and stimulation of the Ras/MAPK signaling pathway and angiogenesis [21].

Adipose tissue, primarily visceral, is associated with a high lipolytic rate, most likely initiated by IL-6 [22].

Lipotoxicity is closely associated with defects in intrahepatic lipolysis due to decreased ATGL/CGI-58 activity, defects in triglyceride export (e.g., defective Apo-B 100, MTTP activity), increased glucokinase activity resulting in increased hepatic DNL, reductions in hepatic mitochondrial/peroxisomal β-oxidation [23].

The balance of reactive oxygen species (ROS) is disrupted under conditions of lipotoxicity. Disruption of the redox balance has been proven to be one of the most important causes of carcinogenesis, proliferation, and metastasis. Elevated levels of ROS activate the cancer cell survival signaling cascade, involving MAPK/ERK1/2, p38, JNK, and PI3K/AKT to activate nuclear factor kappa-light-chain-enhancer of activated B cells (NF-κB), matrix metalloproteinases (MMPs), and vascular endothelial growth factor (VEGF), thereby promoting cancer angiogenesis and metastasis [24]. Adipose tissue also secretes many hormones, among which the effects of adiponectin and leptin have been best studied.

Adiponectin has an oncoprotective impact, but the level of adiponectin is reduced in obese people, especially those with visceral obesity. Reducing the fat mass size in obese people can increase the adiponectin level and, thus, contribute to oncoprotection. The most likely mechanism of adiponectin’s oncoprotection is its positive correlation with insulin sensitivity, as well as its positive physiological influence on glucose and free fatty acid (FA) metabolism [4]. There is also controversy in interpreting adiponectin levels in obese individuals. It is believed that if an oncogenic mutation occurs in an obese individual, an increase in adiponectin levels can also increase the risk for proliferation and metastasis of malignant neoplasms, as well as their resistance to neoadjuvant therapy [4]. This controversy is the subject of further research in the field of oncology based on obesity. A low level of adiponectin, characteristic of obesity, leads to decreased production of sex hormone binding globulin (SHBG), and consequently to increased levels of free estrogen, which is considered as one of the additional reasons for the occurrence of gynecological carcinoma incidence in this population [12].

In obese people, a high level of leptin is a result of leptin resistance, similar to insulin resistance. The carcinogenicity of hyperleptinemia is most likely a consequence of direct stimulation of the STAT3 signaling pathway and stimulation of angiogenesis. Current studies indicate that hyperleptinemia in obese individuals and the overexpression of the leptin receptor are associated with estrogen receptor (ER)-positive lung carcinoma, leading to cellular proliferation through ERα-dependent transcription. Hyperleptinemia in this type of tumor positively correlates with proliferative potential, mitogenicity, infiltrative and metastatic potential, as well as with epithelial–mesenchymal transition (EMT) [4].

In obese people, adipocytes are hypertrophied and show increased expression of aromatase, so obese people face the problem of increased aromatization of androstenedione and testosterone, converting them into estrone and estradiol. Thereby, adipose tissue becomes an additional source of estrogen, which in conditions of obesity turns into oxidized metabolites of estrogen, which, in turn, also leads to oxidative damage to DNA. In obesity, as a negative metabolic indicator, there is a reduced level of SHBG, favoring an increase in free estrogen and the carcinogenesis of tissues sensitive to this hormone. These activities stimulate the mitogenic PI3K/AKT/mammalian target of rapamycin (mTOR) signaling pathway, which is suitable for oncogenesis [7].

In addition, elevated levels of estrogen activate the G-protein-coupled ER 1 (GPER1), which through a ligand-dependent signaling mechanism stimulates endometrial proliferation via activation of the MAPK and AKT signaling pathways [12].

Hypercholesterolemia is one of the most common comorbidities associated with obesity. The effect of hypercholesterolemia on oncogenesis is explained in the following way: It leads to increased free radical production, that results in gene mutations [21], accumulating the lipid droplet (LD) content in porcine oocytes after maturation and upregulating the expression of genes related to mitochondria biogenesis (ACACA, FASN, PPARγ, SREBF1, ATGL, HSL, and PLIN2) [25].

The increased cholesterol level in obese people interferes with hematopoiesis and the differentiation of stem cells, leading to dysregulation of the natural killer cell production. In addition, fat droplets accumulate in dendritic cells in the state of hypercholesterolemia, which prevents adequate presentation of oncoantigens to the immune system [7, 26].

Cholesterol weakens anti-tumor immunity, leading to the depletion of CD8+ T-lymphocytes [27].

Considering this, previous experience shows that statins can lead to cancer cell apoptosis, which releases neoantigens and leads to an adaptive immune response to malignant disease [28].

**Figure 1.**
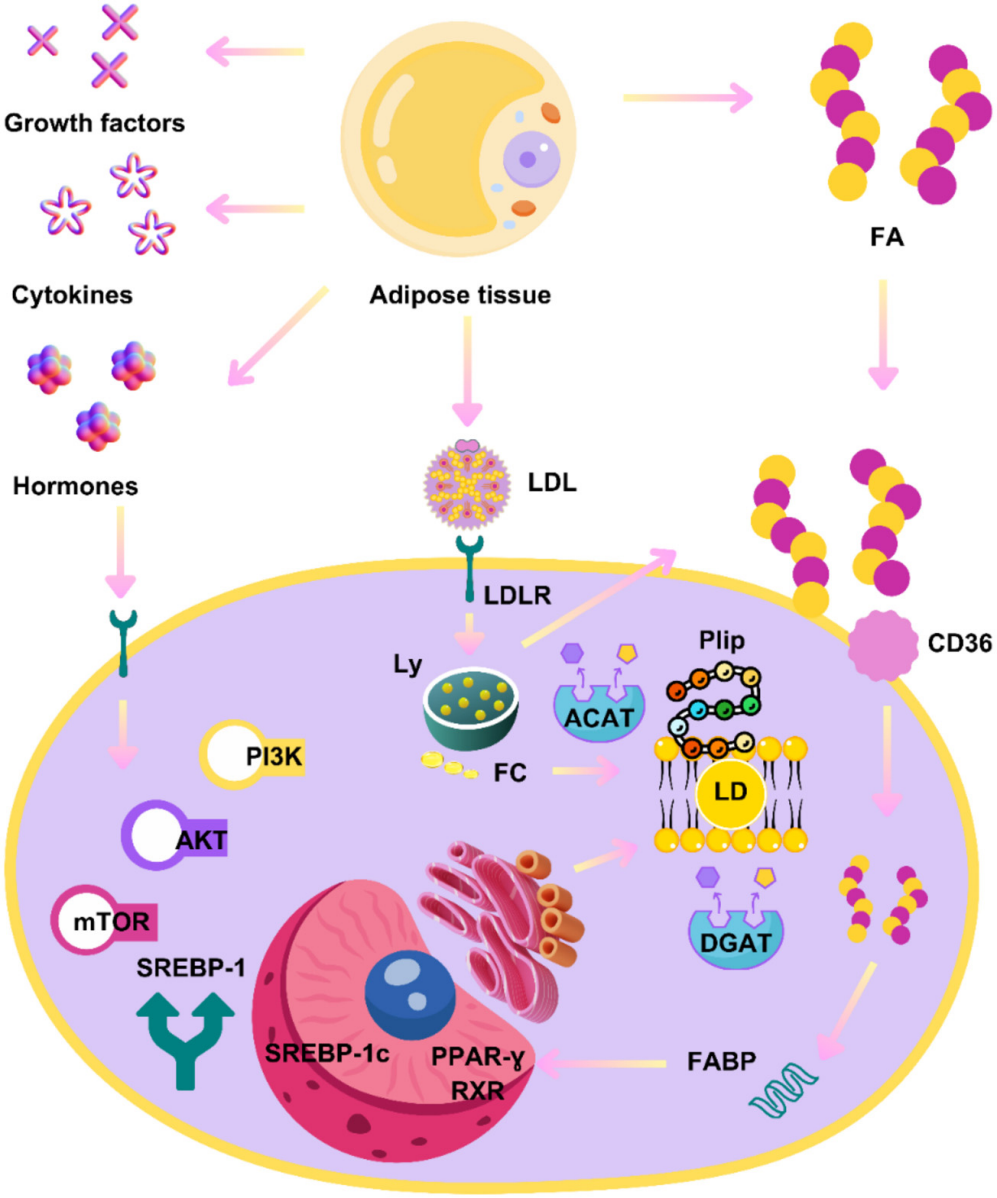
**Lipid droplets, metabolism, and cancer crosstalk.** LD: Lipid droplets; Plip: Perilipin; LDL: Low-density lipoprotein; LDLR: Low-density lipoprotein receptor; FA: Fatty acid; CD36: Fatty acid translocase; Ly: Lysosome; FC: Free cholesterol; ACAT: Cholesterol acyltransferases; DGAT: Diglyceride acyltransferase; FABP: Fatty-acid-binding protein; PPAR-γ: Peroxisome proliferator-activated receptor gamma; RXR: Retinoid X receptor; SREBP: Sterol regulatory elements binding proteins; PI3K: Phosphoinositide 3-kinase; mTOR: Mammalian target of rapamycin. Photo and design software: https://www.canva.com/. Source: https://www.canva.com/.

The intricacy of the relationship between lipid metabolism and cancer is highlighted in the latest findings, emphasizing the significance of LDs as dynamic organelles with multiple functions intertwined in signaling, metabolism, and the production of inflammatory molecules. Specifically, it has been demonstrated that metabolic dysregulation in obesity creates a favorable milieu for ectopic accumulation of LD, which is associated with carcinogenesis, as well as insulin resistance and cardiovascular comorbidities [29, 30]. The structure of LD comprises a phospholipid monolayer membrane and a hydrophobic core consisting of cholesterol esters and triacylglycerols, supplemented by a broad spectrum of cell-specific proteins. Within the cytoplasm, these meticulously structured organelles stand as lipid-rich entities. Perilipin 1–3, belonging to the family of PAT proteins, constitute a group of structural proteins that envelop LD [31, 32]. These organelles are formed in the endoplasmic reticulum (ER). Research has shown that LD, originating in the ER, intricately interplay with all phases of complex carcinogenic cascades, from initiation to promotion and progression [29, 32]. The accumulation of LD in cancer cells involves three crucial processes: increased lipid uptake, modulation of lipolysis, and de novo lipid synthesis. Considering all those mentioned earlier, the complex relationship between carcinogenesis and lipid metabolism can be understood from fundamental steps involving the synthesis of FA as the main structure elements of complex lipids, ensuring anabolic processes crucial in malignant diseases [29, 33]. Various signaling pathways represent key points enabling lipogenesis, leading to the buildup of freshly synthesized LD. Thus, we encountered two critical elements in this process: sterol regulatory elements binding proteins (SREBPs) and mTOR. Upregulated expression of SREBPs leads to the promotion of lipogenesis and subsequent accumulation of LD, resulting in enhanced growth of tumor cells. Additionally, mTOR contributes to those by regulating the accessibility of extracellular nutrients and is closely associated with SREBPs. Previous studies have demonstrated an intriguing link between hyperleptinemia in obesity and mTOR, contributing to an excess of FAs [29, 34–37]. Ultimately, we can conclude that carcinogenesis is tangled with lipolytic and lipogenic enzymes, forming a complex interplay between cancer processes and lipid metabolism [29], as partly illustrated in Figure 1.

The enzyme proprotein convertase subtilisin/kexin type 9 (PCSK9) is essential in the metabolism of cholesterol, which recent tests in the field of oncology have shown to be a potential biomarker of malignant diseases, while tests in the course of studies suggest that PCSK9 inhibitors, which are used in the treatment of hypercholesterolemia, could also be used in the treatment of some forms of cancer. Those drugs negatively affect cell proliferation, invasion, and metastasis of malignant cells. On the other hand, they stimulate apoptosis, anti-tumor immunity, and decrease radioresistance, thus resulting in a better prognosis of malignant disease [38].

Hyperglycemia, whether it full developed or impaired glucose tolerance (IGT), increases the risk of developing endometrial carcinoma 1.8 times [39]. Long-term hyperglycemia impairs cellular respiration, producing superoxide anion, and stimulates VEGF expression, which promotes angiogenesis and tumorigenesis [21]. In 1956, Otto Warburg noticed that malignant cells show increased glycolysis even in aerobic conditions, i.e., increased consumption of glucose and accumulation of a large amount of lactate was observed. The phenomenon, called aerobic glycolysis or the Warburg effect, is observed in almost all malignant cells and is used today for diagnostic purposes as the main characteristic of malignancy when using positron emission tomography (PET) [21].

Through the Warburg effect, under aerobic conditions, glucose-6-phosphate is oxidized, providing ribose-5-phosphate, necessary for nucleotide synthesis, and erythrose-4-phosphate, which participates in amino acid synthesis. This stimulates anabolic activities in tumor tissue, leading to the development and proliferation of malignant tissue [20].

**Figure 2.**
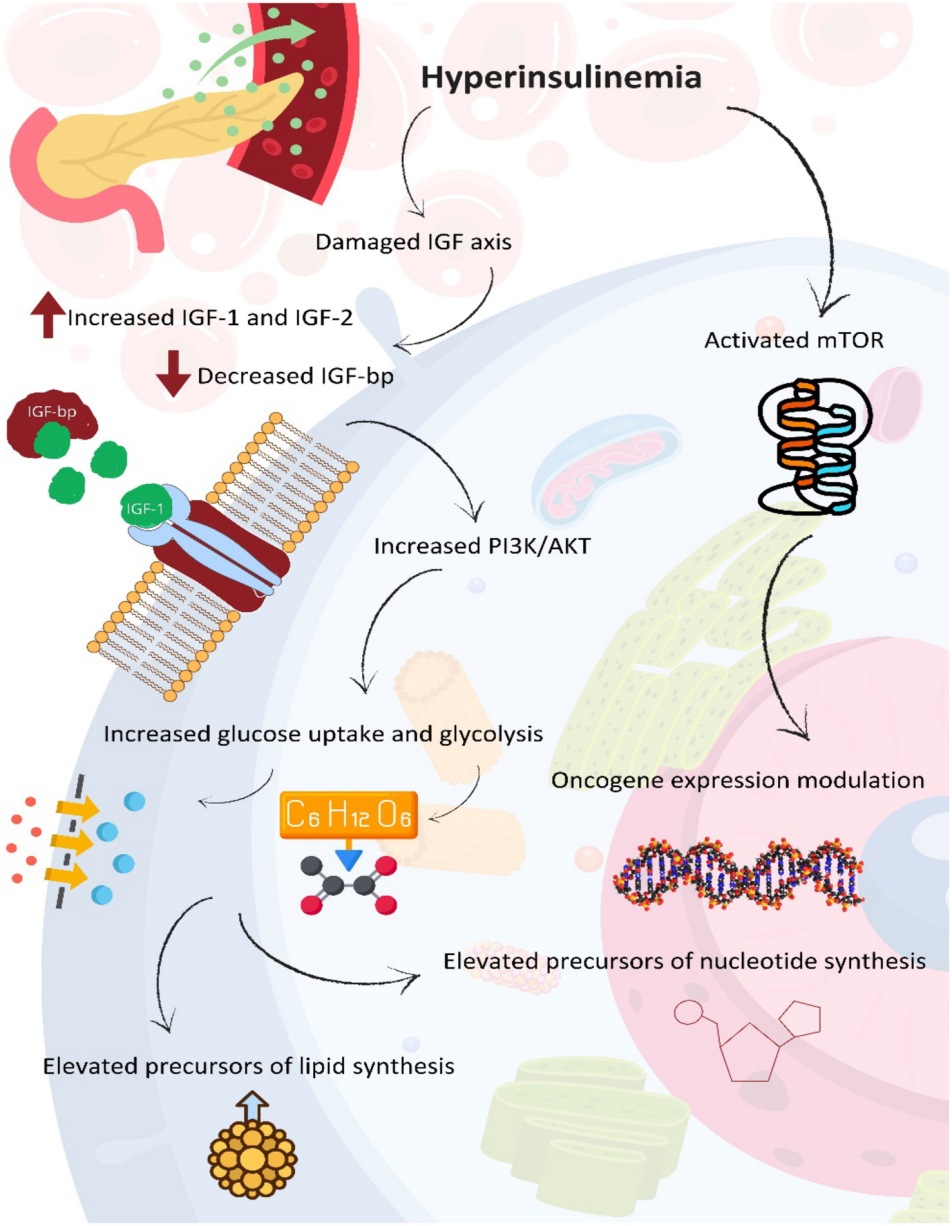
**Signaling cascade from hyperinsulinemia to oncogenesis.** IGF: Insulin-like growth factor; IGF-bp: Insulin-like growth-factor binding protein; PI3K: Phosphoinositide 3-kinase; AKT: Protein kinase B; mTOR: Mammalian target of rapamycin. Photo and design software: https://www.canva.com/. Source: https://www.canva.com/.

Hyperinsulinemia is one of the best-studied metabolic disorders associated with obesity. One of the pathways by which hyperinsulinemia promotes carcinogenesis is described in the chapter on lipotoxicity and is illustrated in Figure 2. On the other hand, hyperinsulinemia also leads to the activation of mTOR and mitogen-activated protein kinase (MARK) signaling pathway, which further leads to the growth of cancer cells and resistance to neoadjuvant therapy [40].

Hypoxia is one of the common disorders associated with obesity. It arises due to insufficient mobility of the diaphragm caused by the accumulation of visceral fat tissue on the one hand, and on the other hand, it is caused by insufficient permeability of the alveolar membrane due to previously described inflammatory cascades associated with obesity. Hypoxia induces distress in the development of the ER, and upregulation of hypoxia-inducible factor 1α (HIF1α), which contributes to aerobic glycolysis and increased glucose uptake, leading to the promotion of malignancy development. This pathway of carcinogenesis is particularly characteristic of the development of breast carcinoma [20].

**Figure 3.**
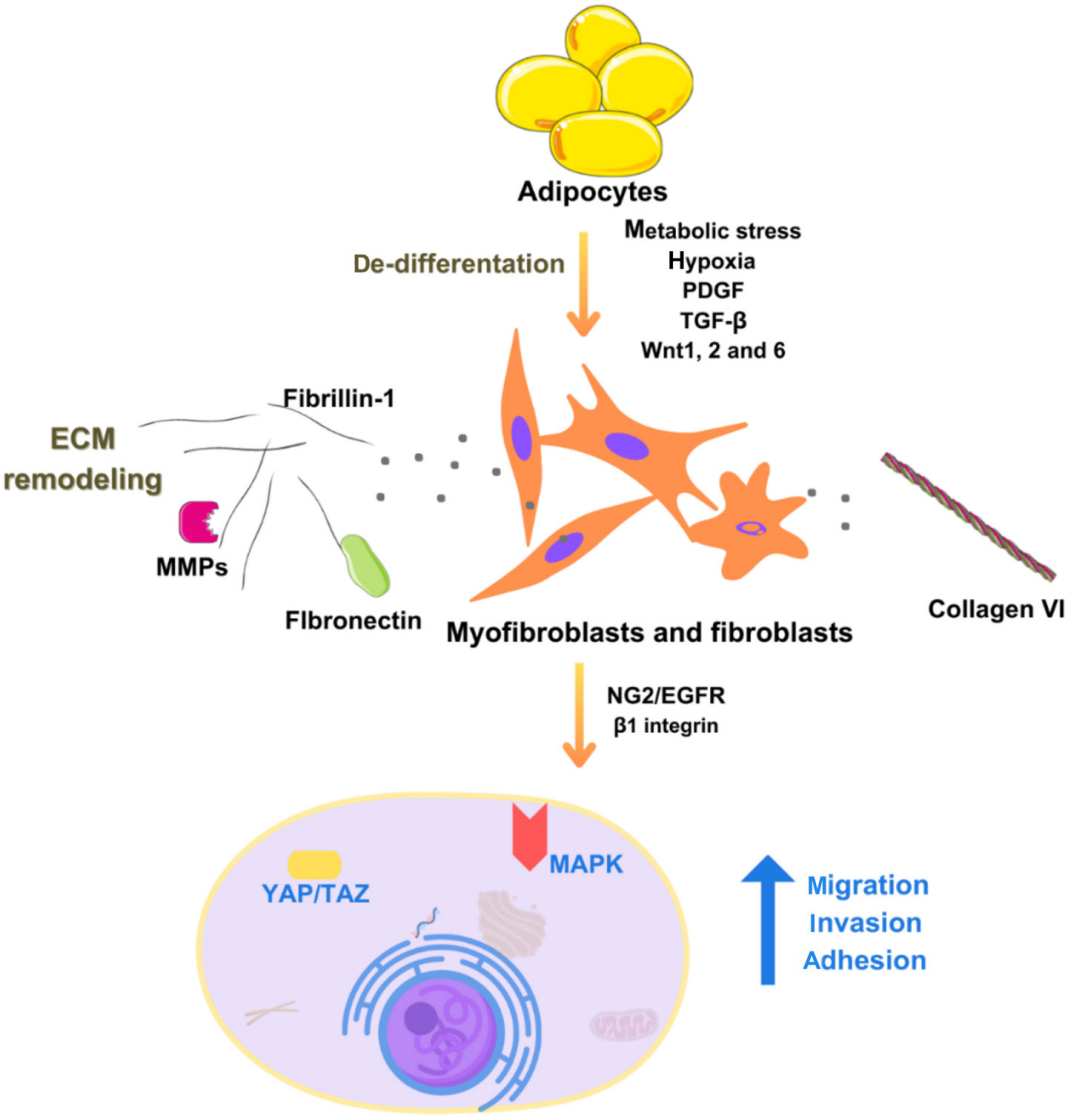
**Impact of adipose tissue extracellular matrix remodeling on obesity-related carcinogenesis**. PDGF: Platelet-derived growth factor; TGF-β: Transforming growth factor beta; Wnt: Wingless/integrated; MMPs: Matrix metalloproteinases; NG2: Neuron glial antigen 2; EGFR: Epidermal growth factor receptor; MAPK: Mitogen-activated protein kinase; YAP: Yes-associated protein; TAZ: Transcriptional coactivator with PDZ-binding motif. Photo and design software: https://www.canva.com/. Source: https://www.canva.com/.

Extracellular matrix (ECM) remodeling and adipose tissue plasticity play a role in carcinogenesis. Adipocytes, in addition to adipocytokines and inflammatory cytokines, also produce components of the ECM, such as collagen I, III, and VI, MMPs, fibronectin, elastin, and fibrillin-1. In obese individuals, the occurrence of more pronounced collagen deposits has been proven, leading to fibrosis. Consequently, tissue fibrosis and interstitial stiffness initiate tumor migration/invasion and poor survival in patients with malignancies [41]. Myofibroblasts/fibroblasts, activated by adipocytes, but also the processes of inflammation and macrophage infiltration, are directly responsible for the onset of fibrosis in adipose tissue. This mechanism has been well studied and published, particularly in the case of breast carcinoma in obese women [41]. Increased stiffness of visceral adipose tissue is caused by the activation of transcription factors YAP/TAZ, which among other things, also promote tumor growth. The other mechanism involves the NG2/EGFR and beta1 integrin pathways, which activate the MAPK signaling cascade (Figure 3) [41, 42].

## Treatment options for metabolic dysregulation in patients with gynecological malignancies

Apart from the difficulties related to the examination and operative treatment of obese patients (specific beds for the examination and operation of obese patients, instruments adapted to obese patients, greater distance between the vulva and the cervix, the accumulation of fatty tissue on the front abdominal wall and around the internal organs), the challenges are also reflected in the more significant cytotoxicity and pronounced resistance to neoadjuvant therapy [43].

**Figure 4.**
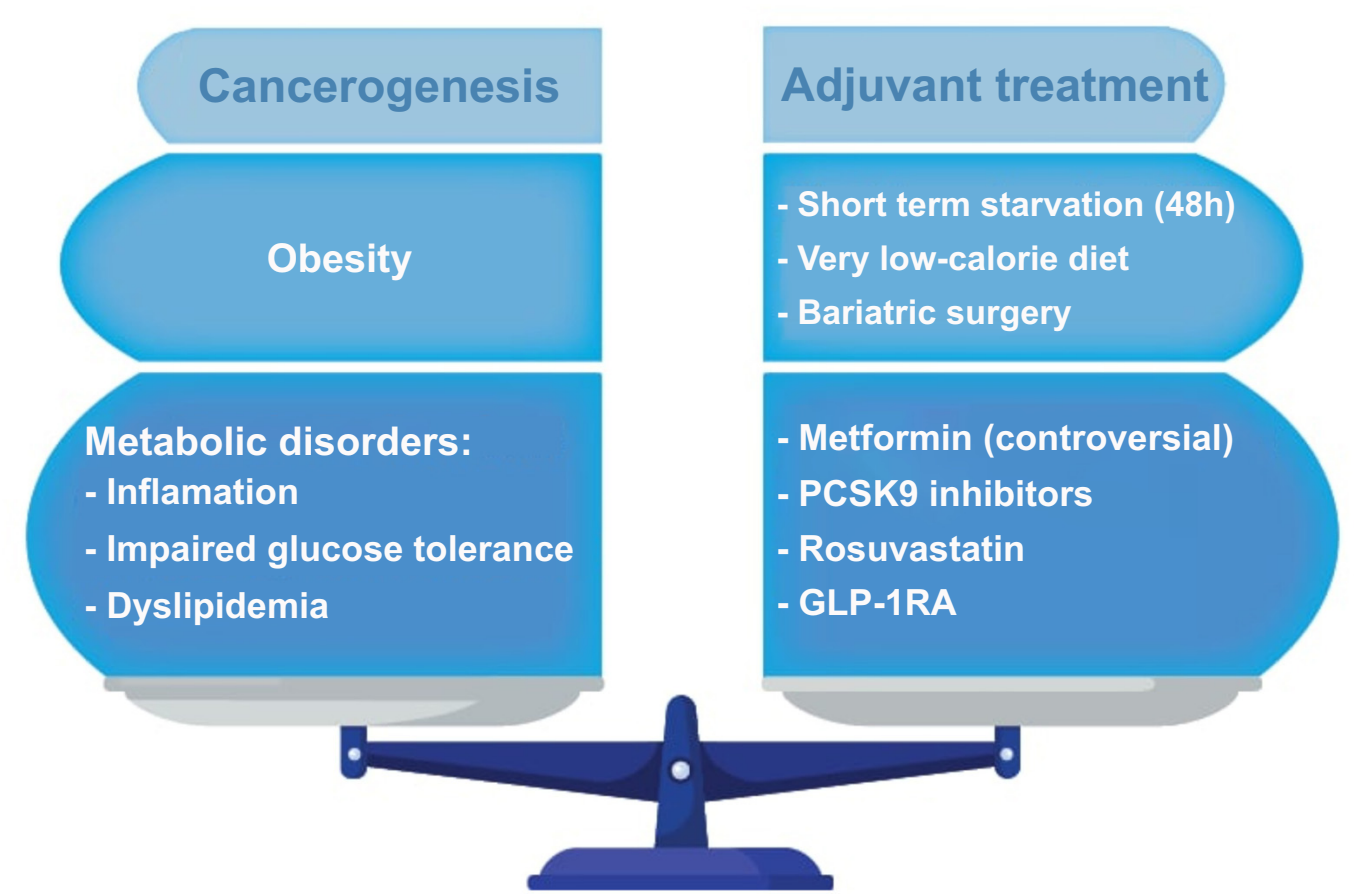
**Metabolic disorders, tumorogenesis, and adjuvant treatment options in obese women.** PCSK9: Proprotein convertase subtilsin-kexin type 9; GLP-1RA: Glucagon-like peptide-1 receptor agonists. Photo and design software: https://www.adobe.com/products/illustrator.html and Microsoft Office – Word 2021.

Taking into account the facts mentioned in the previous paragraphs, in order to address this problem in obese patients, it is necessary to carry out weight reduction (with medical nutritional therapy, medication, or bariatric surgery), use statins and other hypolipidemic drugs in dyslipidemias, improve glycoregulation by using insulin sensitizers (Figure 4) [43].

### Medical nutritional therapy

As already described in this study, the association of gynecological cancers with obesity suggests that dietary habits and lifestyle changes would benefit public health, aiming to prevent obesity and its comorbidities [12]. This could be achieved through organized campaigns via mass media, social networks, and improved health education starting from a young age.

Reducing diets are reserved for treating obese individuals without malignancies or for preventing obesity. Although the Mediterranean diet is often emphasized, modern findings suggest that it reduces cardiovascular risk but does not lead to a rapid decline in body mass. It is necessary to achieve an energy deficit when implementing a diet, which is why low-calorie diets (LCDs) and very low-calorie diets (VLCDs) (400–800 kcal) are described [44].

LCDs involve a caloric deficit diet, usually calculated based on the current needs for an individual’s ideal body weight minus 500 kcal. In outpatient settings, it is recommended that the caloric intake not fall below the basal metabolic rate and not below 1200 kcal.

VLCDs are implemented briefly under controlled conditions, often as a precursor to bariatric surgery. The daily energy intake is 400–800 kcal, with 30–50 grams of carbohydrates, 20–40 grams of fat, and 1.2–1.4 grams of protein per kilogram of body weight. This type of diet reduces the volume of a fatty liver, visceral fat mass, and perioperative complications in obese individuals [45].

It has been proven in an animal model that chronic caloric reduction lowers the incidence and mortality of cancer [46].

These findings are, for now, limited to the human population, considering the possibility of developing tumor cachexia. However, recently, studies have shown that cycles of short-term starvation (STS) can increase the sensitivity of cancer cells to cytoreductive therapy [43].

Pateras et al. concluded that intermittent fasting for 48 h in combination with chemotherapy significantly reduces the potential of tumors to grow and metastasize. This phenomenon is explained by cancer cells’ dependence on nutrients and tumor cells’ insufficient adaptability to oxidative stress [43]. Namely, the VLCD triggers the production of ketone bodies, which induce the synthesis of adenosine monophosphate-activated protein kinase (AMPK), leading to an anti-oxidative, anti-inflammatory effect, improvement of mitochondrial functions, and the ability of cells to self-regenerate. However, this mechanism occurs in healthy cells, while tumor cells do not have this adaptive mechanism [8].

It is known that cholesterol metabolism is crucial for cancer development through an immunomodulatory mechanism in the cancer’s microenvironment, caused by suppressing the activity of type I natural killer T (NKT) cells [47].

### Hypolipidemic drugs

It is shown that statins are effective in normalizing the function of NKT cells when used to reduce LDL cholesterol. The study was conducted on an animal model of hepatocellular carcinoma, but the normalization of NKT lymphocytes was observed in both hepatocytes and circulation. This effect has been demonstrated for rosuvastatin [48].

Evolocumab and alirocumab, monoclonal antibody PCSK9 inhibitors, increase LDL receptor expression on hepatocytes. This mechanism leads to the utilization of LDL cholesterol in hepatocytes, thereby reducing the availability of cholesterol particles necessary for metabolizing tumor particles and producing steroid hormones. Increased LDL receptor expression ultimately affects the growth rate of gynecological cancers [49, 50].

In a human model of hormone-dependent breast cancer, even natural PCSK9 inhibitors, such as Pseurotin A (PS) (isolated from the culture of the fungus *Pseudeurotium ovalis*), have been shown to have a beneficial effect on suppressing tumorogenesis and tumor growth [49]. The role of PCSK9 synthesis inhibitors (siRNA) on cancer behavior remains undefined for future research, given that this type of drug is assumed not to have the same systemic effect as PCSK9 inhibitory antibodies [49].

### Medications affecting glucose metabolism

Metformin achieves its role as an insulin sensitizer through the activation of the central energy sensor AMPK. Activated AMPK downregulates PI3K/AKT/mTOR signaling, directly targeting respiratory complex I of the electron transport chain in mitochondria, reducing energy supply, and activating an integrated stress response involving ROS and DNA damage. They induce the expression of regulated in DNA damage 1 (REDD1) [51].

It was considered that metformin has a beneficial effect on cancer prevention and better outcomes for patients with malignancy who use metformin since 2005, in 2012, there were doubts about this issue and the assumption that metformin can influence the outcome of the disease only in the diabetic population. After 2022, the American Diabetes Association (ADA) does not provide clear recommendations for using metformin as adjuvant therapy, and numerous clinical randomized studies are ongoing, which should shed light on this dilemma [52].

Signals from oncologists in 2022 suggest that metformin can be used, and there is a clear benefit in T2D and HER2-positive breast cancer patients. On the other hand, there is a clear need for new clinical randomized studies focused on HER2-positive breast cancer disease, which would clarify the dilemma and the possibility of individualizing metformin therapy in individuals with malignancies [51]. Regarding the treatment of early endometrial carcinoma, the results of the meta-analysis by Adamyan et al. suggest that adding metformin to progestin therapy improves remission and pregnancy rates in atypical endometrial hyperplasia. Still, it does not significantly affect relapse or live birth rates [53].

Although there are no recommendations from gynecological oncology regarding gynecological malignancies and the benefit of using GLP-1 analogs in these diseases, the results of using GLP-1 analogs in women with polycystic ovary syndrome are well known. Namely, in this population, it was proven that GLP-1 analogs with or without metformin lead to a significant reduction in BMI and the proportion of fat tissue in the body composition, with benefits on insulin resistance indicators [52]. Taking into account the statements of Yu et al. and knowing that BMI and percentage of fat mass positively correlate with the occurrence of endometrial cancer, cervical cancer, and some histological forms of ovarian cancer opens a wide field for research and publication of the experiences of applying GLP-1 analogs as adjuvant therapy for gynecological malignancies.

### Bariatric surgery

The most effective method for treating obesity is bariatric surgery, which can achieve sustainable weight loss of over 25%, and it is undoubtedly superior to medical therapy, where the effect of reduction achieved through clinical randomized studies is around 10%. The sustainability of the results remains to be discovered. Bariatric surgery is indicated for individuals with BMI >35 kg/m^2^, regardless of the presence or severity of co-morbidities, and for individuals with a BMI of 30–34.9 kg/m^2^ and metabolic disease.

BMI should be adjusted in the Asian population, where a BMI >25 kg/m^2^ suggests clinical obesity and individuals with a BMI >27.5 kg/m^2^ should be offered bariatric surgery. Selected children and adolescents should be considered [54].

Aminian et al. published a study involving 30,318 obese patients (5053 treated with bariatric surgery and 25,265 treated conservatively with a reduced-calorie nutritional diet and medication). Over an average follow-up period of 6.1 years, all obesity-related malignancies were observed in both groups. Breast cancer and endometrial cancer emerged as the two most common malignancies. This study’s results indicate a significant difference in weight loss between the two groups under investigation (the group treated with bariatric surgery had an average weight loss of 27.5 kg, while the conservative treatment group achieved a loss of 2.7 kg). The cumulative incidence of the observed cancers was lower in the bariatric surgery-treated group. The cumulative incidence in patients treated with bariatric surgery depended on the achieved weight loss in quartiles. Individuals with a weight loss of less than 24% had the highest incidence of cancer, while the best prevention of malignancies was achieved in the group that lost more than 39% of their initial body weight [55].

The reduction in the incidence of malignancies after bariatric surgery likely occurs through a reduction in the proportion of dysfunctional adipose tissue, thereby reducing inflammation, reducing hyperinsulinemia and IGF-1 levels, as well as modulating, or balancing the levels of sex hormones and adipokines, bringing their levels into desirable ratios characteristic of a healthy individual, and reducing markers of proliferation and oncogenic signaling pathways described in earlier chapters [56, 57].

This effect has been particularly well studied in the case of endometrial cancer, and these results may have important implications for the screening, prevention, and treatment of this disease. MacKintosh et al. conducted a study demonstrating that in women with stage II and III obesity (BMI >35 kg/m^2^ and BMI >40 kg/m^2^), after bariatric surgery, the median Ki-67 score was 15.1% lower at two months and 15.8% lower at 12 months after surgery. Additionally, compared to baseline, glandular phosphorylated AKT (pAKT) was 15% lower at two months and 11.7% lower at 12 months after bariatric surgery, and a statistically significant decrease in the expression of ER and PR was observed after bariatric surgery [58].

As the authors note, bariatric surgery, with consequent weight loss, changes the proinflammatory milieu suitable for endometrial carcinogenesis [58].

Given this knowledge about the rapid change in the proinflammatory and carcinogenic milieu after bariatric surgery, and since endometrial cancer, among gynecological cancers, is most commonly associated with obesity, bariatric surgery should certainly be recommended to women with stage II and III obesity to prevent this malignancy. Preparation for bariatric surgery is a complex process that requires a team of experts, including endocrinologists, cardiologists, psychologists, and surgeons. For this reason, it is necessary to ensure fast and easy communication between gynecologists and the mentioned specialties and to provide patients with easy access through the healthcare network.

### Physical activity

Physical activity, or exercise, is recommended, given evidence that it positively affects the prevention of malignancies and the survival of individuals with cancer [59]. Exercise is an indispensable form of treatment for individuals diagnosed with cancer during therapy and in the recovery period. It must be individualized according to the person’s physical performance, nutritional status, degree of malignant disease, and current symptoms. Any form of activity is recommended, from passive exercises in bed and aerobic exercises to resistance exercises [60]. Exercise contributes to improving the metabolic environment mainly through insulin-dependent pathways, which, as we have already described, by activating numerous signaling pathways, contribute to oncogenesis and the biological behavior of cancer [61].

## Conclusion

Knowing the consequences of metabolic dysregulation, the future in the prevention and treatment of all malignancies, including gynecological malignancies, would undoubtedly require, in addition to surgical and adjuvant therapy, personalized therapy concerning obesity in terms of weight reduction or bariatric surgery and nutritional and medical influence on the metabolic reprogramming of the tumor microenvironment, which is proven to participate in the etiopathogenesis of malignancy and compromise the outcome of treatment.
